# Inducible and Deterministic Forward Programming of Human Pluripotent Stem Cells into Neurons, Skeletal Myocytes, and Oligodendrocytes

**DOI:** 10.1016/j.stemcr.2017.02.016

**Published:** 2017-03-23

**Authors:** Matthias Pawlowski, Daniel Ortmann, Alessandro Bertero, Joana M. Tavares, Roger A. Pedersen, Ludovic Vallier, Mark R.N. Kotter

**Affiliations:** 1 Anne McLaren Laboratory, Wellcome Trust-MRC Stem Cell Institute, University of Cambridge, Cambridge CB2 0SZ, UK; 2 Department of Clinical Neuroscience, University of Cambridge, Cambridge CB2 0QQ, UK; 3 Department of Surgery, University of Cambridge, Cambridge CB2 0QQ, UK; 4 Wellcome Trust Sanger Institute, Hinxton, Cambridge CB10 1SA, UK; 5 Department of Paediatrics, University of Cambridge, Cambridge, CB2 0QQ, UK

**Keywords:** human pluripotent stem cells, reprogramming, skeletal myocytes, oligodendrocyte progenitor cells, neurons

## Abstract

The isolation or in vitro derivation of many human cell types remains challenging and inefficient. Direct conversion of human pluripotent stem cells (hPSCs) by forced expression of transcription factors provides a potential alternative. However, deficient inducible gene expression in hPSCs has compromised efficiencies of forward programming approaches. We have systematically optimized inducible gene expression in hPSCs using a dual genomic safe harbor gene-targeting strategy. This approach provides a powerful platform for the generation of human cell types by forward programming. We report robust and deterministic reprogramming of hPSCs into neurons and functional skeletal myocytes. Finally, we present a forward programming strategy for rapid and highly efficient generation of human oligodendrocytes.

## Highlights

• Dual genomic safe harbor targeting of the Tet-ON system
• Optimized inducible transgene expression in human pluripotent stem cells
• Deterministic forward programming into neurons, myocytes, and oligodendrocytes

## Graphical Abstract

**Figure:**
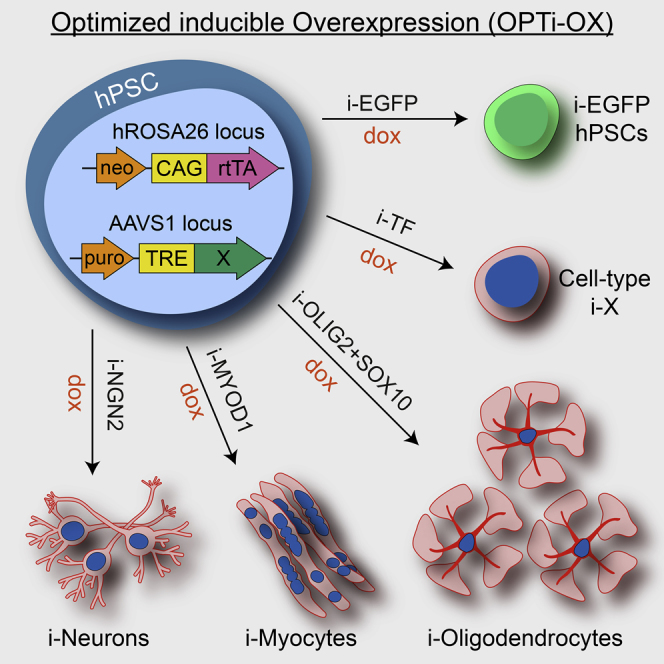

## Teaser

In this article, Pawlowski and colleagues report a dual genomic safe harbor targeting approach for optimized inducible transgene expression in human pluripotent stem cells (hPSCs). The optimized inducible expression of reprogramming factors in hPSCs enables deterministic forward programming into mature cell types. This is exemplified by the rapid, single-step generation of neurons, skeletal myocytes, and oligodendrocytes.

## Introduction

Despite major efforts to develop robust protocols for scalable generation of human cell types from easily accessible and renewable sources, the differentiation of human pluripotent stem cells (hPSCs) into specific cell types often remains cumbersome, lengthy, and difficult to reproduce. Moreover, the recapitulation of developmental stages in vitro yields fetal cells that often do not reach full maturation (Cohen and Melton, 2011). More recently, forced expression of lineage-specific master regulators resulting in direct reprogramming of somatic cell types has provided an efficient alternative to directed differentiation (Huang et al., 2014, Ieda et al., 2010, Zhou et al., 2008). In particular, the direct conversion of hPSCs, termed forward programming (Moreau et al., 2016), combines the advantages of hPSC differentiation and direct cellular reprogramming, enabling scalable and rapid generation of human cell types (Zhang et al., 2013).

Currently available forward programming protocols are largely based on lentiviral transduction of hPSCs, which results in variegated expression or complete silencing of transgenes (Darabi et al., 2012, Smith et al., 2008). Additional purification steps are usually necessary for enriching the desired cell type. Lentiviral approaches randomly insert transgenes into the genome bearing the risk of unwanted interference with the endogenous transcriptional program. Therefore, refinements to the current forward programming approaches are desirable.

As the result of a systematic effort to optimize gene expression in hPSCs, we arrived at a robust hPSC forward programming platform by targeting all components of the Tet-ON system required for inducible expression of transcription factors into genomic safe harbor sites (GSHs) (Sadelain et al., 2012). The Tet-ON system consists of two components: a constitutively expressed transcriptional activator protein responsive to doxycycline (dox) (reverse tetracycline transactivator [rtTA]), and an inducible promoter regulated by rtTA (Tet-responsive element) that drives expression of the transgene (Baron and Bujard, 2000). Previous GSH-targeting strategies of the Tet-ON system relied on introducing both elements into the AAVS1 GSH of hPSCs, either separately (Hockemeyer et al., 2009), or together (using an all-in-one Tet-ON vector) (Ordovás et al., 2015, Qian et al., 2014). Compared with these designs, we reasoned that targeting each of the two elements of the Tet-ON system into a different GSH would have several advantages: inducible overexpression based on dual GSH targeting would not be affected by promoter interference between the two transgenes (Baron and Bujard, 2000), while homozygous GSH targeting would maximize the number of safely targeted transgene copies. Moreover, the larger cargo capacity in each of the transgenes would permit increased flexibility for transgene design, thus allowing the insertion of large reprogramming cassettes.

Here we show that an optimized approach based on dual GSH targeting of Tet-ON-controlled transgenes results in homogeneous, controllable, and extremely high expression of inducible transgenes in hPSCs. Application of the optimized overexpression platform enabled us to develop rapid and deterministic forward programming protocols for mature human cell types.

## Results

### Development of an Optimized Inducible Transgene Overexpression Method by Dual GSH Targeting

**Figure 1:**
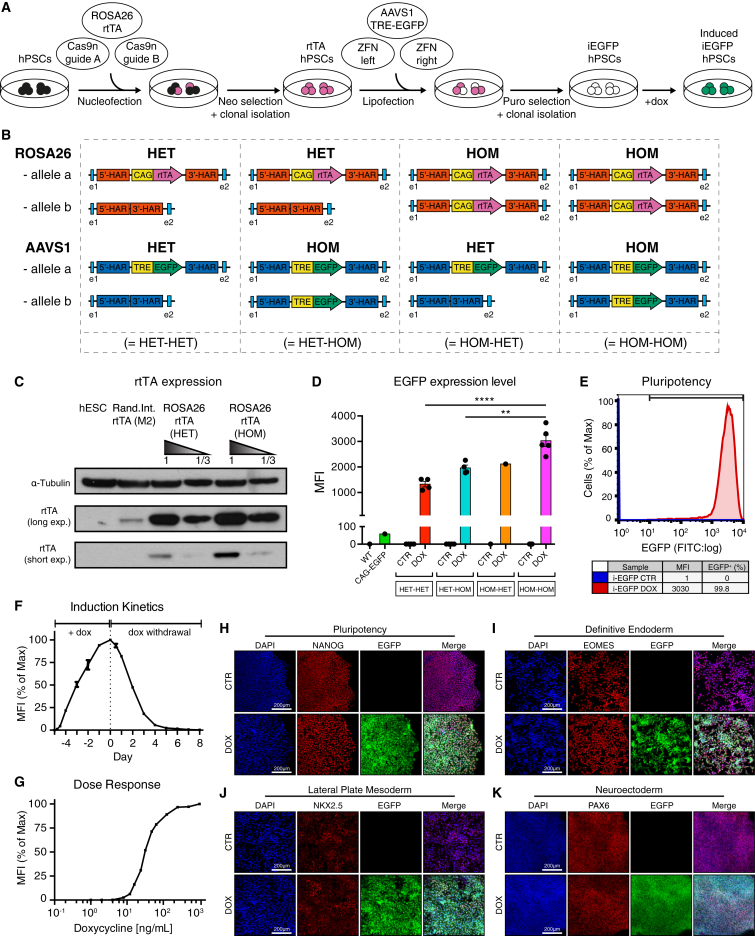
Development of an Optimized Inducible Gene Overexpression System (A) Workflow for targeting the hROSA26 and AAVS1 loci with the Tet-ON system in hPSCs for inducible EGFP expression (i-EGFP). Cas9n, D10A nickase mutant Cas9 endonuclease; ZFN, zinc-finger nucleases; rtTA, reverse tetracycline transactivator; TRE, Tet-responsive element. (B) Schematic of the four outcomes following generation of dual GSH-targeted inducible EGFP hESCs: clonal lines were categorized based on the number of successfully targeted alleles of the hROSA26 and AAVS1 loci. (C) Detection of the rtTA protein by western blot in heterozygous (HET) and homozygous (HOM) hROSA26-CAG-rtTA hESCs. Homozygous targeting results in increased rtTA protein expression. hESCs with random integration of a second-generation rtTA (M2-rtTA) and wild-type hESCs are shown as positive and negative reference. α-Tubulin, loading control. (D) Median fluorescent intensity (MFI) of EGFP expression in the various dual GSH-targeted i-EGFP hESCs described in (B). Cells were analyzed by flow cytometry in non-induced conditions (CTR) or following 5 days of dox. AAVS1-CAG-EGFP and wild-type (WT) hESCs were included for comparison. Statistical analysis of dox-treated groups demonstrated that EGFP levels were highest in double-homozygous clones (each data point, n = 1–5, represents a clonal line; mean ± SEM; one-way ANOVA with post hoc Dunnett's test; ^∗∗^p < 0.01, ^∗∗∗∗^p < 0.0001). (E) Flow cytometry of EGFP OPTi-OX hESCs after 5 days of dox treatment. Non-induced cells were included as negative control. (F and G) EGFP induction and rescue kinetics (F) and dox dose-response (G) in EGFP OPTi-OX hESCs detected by flow cytometry (n = 2 biological replicates; mean ± SEM; all values normalized to the maximum fluorescence intensity after 5 days of dox). (H–K) Immunocytochemistry (ICC) for lineage-specific markers in undifferentiated EGFP OPTi-OX hESCs and following differentiation into the germ layers.

To optimize inducible transgene overexpression from GSHs, we generated human embryonic stem cells (hESCs) with inducible EGFP (i-EGFP) expression. Initially, we tested four different designs (Figure S1A). These comprised two all-in-one targeting constructs in which both rtTA and i-EGFP expression cassettes (third-generation Tet-ON system) were inserted into the same allele of the AAVS1 GSH locus. In these constructs, the rtTA expression was under the control of either an EF1α or CAG promoter. The other two transgene designs were based on spatial separation of the activator and responder into two distinct GSHs (Figure S1A). For this purpose, we sequentially targeted the rtTA cassette into the human ROSA26 GSH (Bertero et al., 2016) and an i-EGFP transgene into the AAVS1 GSH (Figures 1A and S1A–S1E) (Hockemeyer et al., 2009). Robust and homogeneous inducible transgene expression was achieved only when the dual GSH approach and a CAG promoter for rtTA expression was used (Figure S1A). Importantly, the dual GSH-targeting approach was highly efficient (Table S1), and did not affect hESC self-renewal or differentiation (Figures 1H–1K).

Using the CAG promoter-based dual GSH-targeting approach, we selected clonal lines that carried either one or two copies of each of the transgenes (Figure 1B), and observed that homozygous targeting of both elements allowed maximal inducible overexpression (Figures 1C, 1D, S1E, and S1F). Under these conditions, EGFP expression was induced homogeneously in all cells, consistent across multiple clones, and more than 50-fold higher compared with EGFP expression via the strong constitutive CAG promoter (Figures 1D, 1E, S1E, and S1F). Maximal EGFP levels were reached approximately 4 days after induction, and expression was quickly reversed upon dox withdrawal (Figure 1F). Moreover, EGFP expression could be titrated by adjusting the dose of dox (Figure 1G). I-EGFP expression was highly efficient in hESCs (Figures 1E and 1H), and during germ layer differentiation (Figures 1I–1K and S1G). There was no detectable background expression of EGFP in the absence of dox (Figures 1D, 1E, and S1G).

Taken together, these results established that homozygous dual GSH targeting of the Tet-ON system is a powerful strategy for homogeneous and controllable expression of inducible transgenes in hPSCs and their derivatives. We will refer to this platform as “OPTi-OX” (optimized inducible overexpression).

### Human Induced Neurons

To test the applicability of the OPTi-OX platform for forward programming of hPSCs into mature cell types, we first chose to generate excitatory cortical neurons, as previous studies demonstrated that these can be readily derived by lentiviral overexpression of pro-neuronal transcription factors in hPSCs (Zhang et al., 2013).

**Figure 2:**
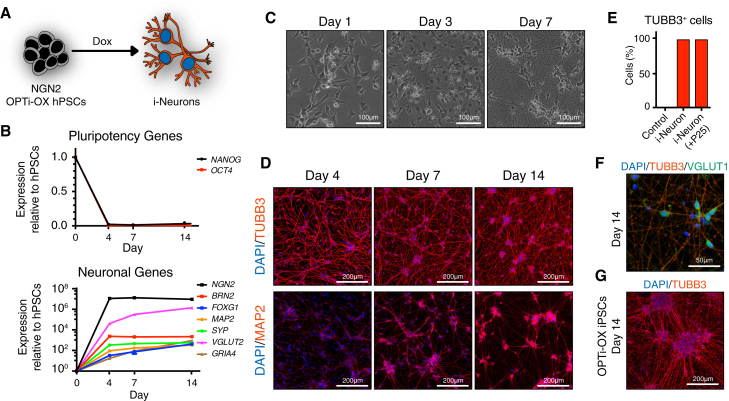
Forward Programming of hPSCs into Neurons (A) Experimental approach for conversion of NGN2 OPTi-OX hPSCs into i-Neurons. (B) Time course of i-Neuron generation from hESCs by qPCR demonstrating the expression pattern of pluripotency factors (*OCT4* and *NANOG*), pan-neuronal (*MAP2* and *SYP*), forebrain (*BRN2*, *FOXG1*), and glutamatergic neuronal marker genes (*VGlut2*, *GRIA4*) (n = 3 biological replicates; mean ± SEM; relative to *PBGD* and normalized to pluripotency). (C) Phase contrast images illustrating the morphological changes during i-Neuron generation (a corresponding time-lapse is shown in Movie S1). (D) ICC for the pan-neuronal marker proteins βIII-tubulin (TUBB3) and microtubule-associated protein 2 (MAP2) during the generation of i-Neurons. (E) Quantification of βIII-tubulin-positive neuronal cells by ICC after 1 week of induction. Undifferentiated cells were used as negative control, and numbers are reported for i-Neuron generation in newly isolated NGN2 OPTi-OX hESCs and after 25 passages. (F and G) ICC for neuronal markers in i-Neurons 14 days after induction.

To this end, we generated NGN2 OPTi-OX hPSCs (Figure 2A; Table S1), and treated them with dox in chemically defined neuronal culture medium (Zhang et al., 2013). Induction of NGN2 expression (Figure S2A) resulted in downregulation of pluripotency factors and initiation of the neuronal transcriptional program (Figure 2B). Dox-treated cells extended neuronal processes as early as 3 days post induction. After 1 week, all cells displayed a neuronal morphology and expressed the pan-neuronal markers βIII-tubulin and MAP2 (Figures 2C–2E and Movie S1). At this stage, induced neurons (i-Neurons) showed strong expression of forebrain markers *BRN2* and *FOXG1*, and of glutamatergic neuronal genes *GRIA4, VGLUT1*, and *VGLUT2* (Figures 2B and 2F), indicative of an excitatory cortical neuronal identity of the forward-programmed cells, consistent with previous reports (Zhang et al., 2013). Short pulses of dox treatment for 4 days or longer sufficed for complete conversion, and converted cells did not rely on continuous transgene expression (Figures S2B and S2C). Importantly, we did not observe any reduction in the efficiency of generating i-Neurons over extended culture periods of the inducible hESCs (>25 passages, Figure 2E). Finally, we confirmed the applicability of the NGN2 OPTi-OX system in hiPSCs (Figure 2G). Collectively, these results demonstrated that OPTi-OX enables robust and rapid forward programming of hPSCs into cortical neurons.

### Human Induced Skeletal Myocytes

To further explore the potential of OPTi-OX for forward programming of hPSCs, we focused on generating human skeletal myocytes. Existing protocols for the directed differentiation of skeletal myocytes from hPSCs are difficult, time consuming, and result in low and variable yields (Chal et al., 2015). On the other hand, myogenic transdifferentiation has been achieved by overexpressing the transcription factor MYOD1 in somatic cell types, but the ability of hPSCs to undergo MYOD1-induced forward programming is a matter of debate (Abujarour et al., 2014, Albini et al., 2013, Tanaka et al., 2013).

**Figure 3:**
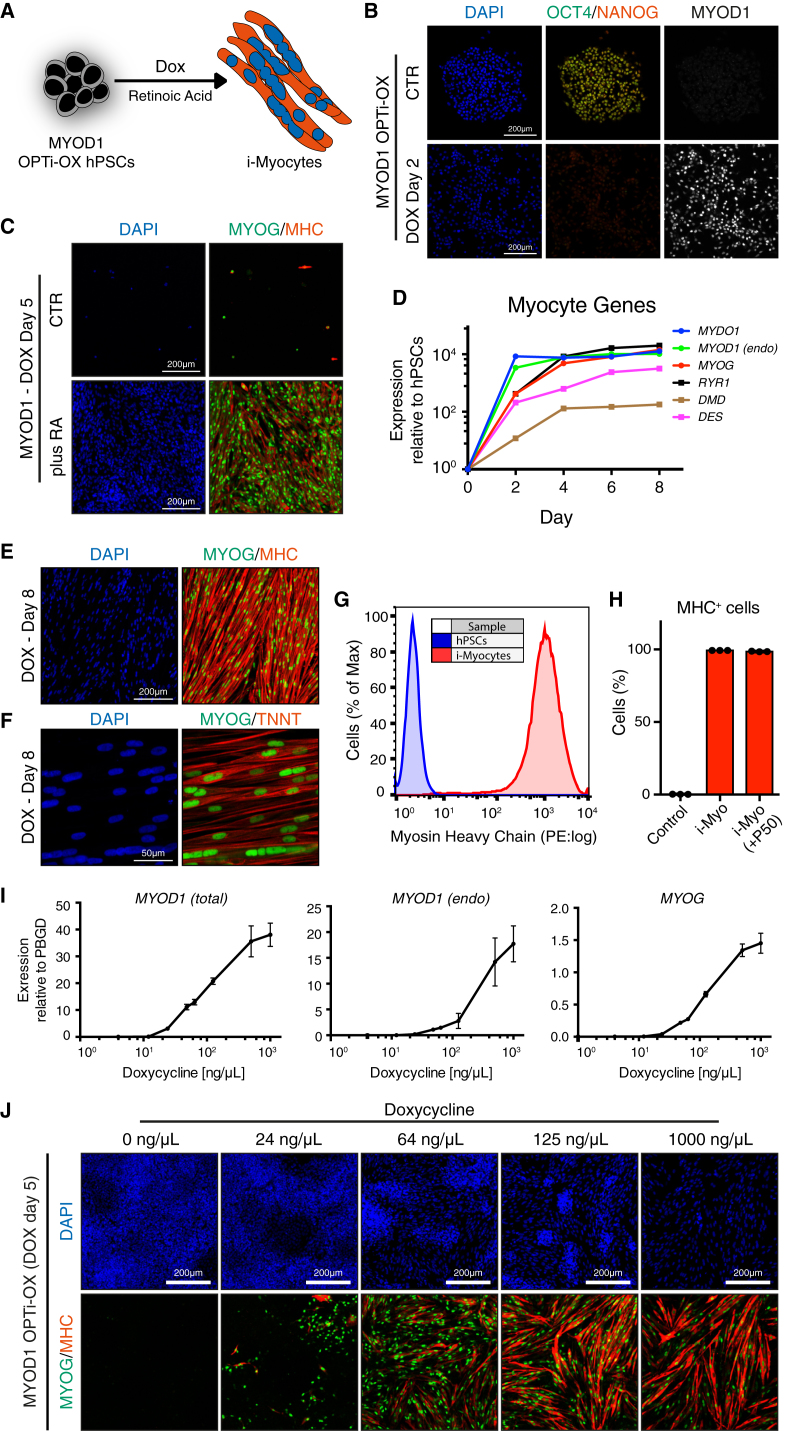
Forward Programming of hPSCs into Skeletal Myocytes (A) Experimental approach for rapid single-step conversion of MYOD1 OPTi-OX hPSCs into skeletal myocytes (i-Myocytes) following treatment with dox and RA. (B) Representative ICC for MYOD1 before (CTR) and after induction with dox. This demonstrates homogeneous induction of transgene expression, paralleled by downregulation of the pluripotency factors NANOG and OCT4. (C) Effect of RA on myocyte forward programming compared with otherwise identical control (CTR) induction conditions (see Figure S2B for the entire signaling molecule screen). (D) qPCR of the temporal expression pattern of pluripotency factors (top panel) and myocyte marker genes during i-Myocyte generation (n = 3 biological replicates, mean ± SEM; relative to *PBGD* and normalized to pluripotency). (E and F) ICC for skeletal myocyte markers in i-Myocytes. (G and H) Quantification of MHC-positive cells by flow cytometry 10 days after induction. Undifferentiated cells were used as negative control, and figures are reported for i-Myocyte generation in newly isolated MYOD1 OPTi-OX hESCs, or in the same cells following 50 passages (+P50) (n = 3 biological replicates; mean ± SEM). (I) qPCR for total *MYOD1*, endogenous *MYOD1*, and *MYOG* 2 days post induction with different dox concentrations (n = 3 biological replicates; mean ± SEM). (J) ICC for myogenin and myosin heavy chain following 5 days of induction with different dox concentrations. Non-converted, proliferative cell clusters appeared when the dox concentration was lowered to 0.125 μg/mL. Further reduction of dox resulted in an increase in non-myocyte cell populations.

We therefore generated MYOD1 OPTi-OX hPSCs (Figures 3A and 3B and Table S1). However, induction of MYOD1 expression following dox treatment resulted in cell death within 3–5 days, regardless of the culture medium used (data not shown). These findings demonstrated that MYOD1 overexpression alone was not sufficient to drive myogenesis in hPSCs, in agreement with the postulated existence of epigenetic barriers preventing forced myogenesis (Albini et al., 2013).

Cellular reprogramming strategies can be enhanced by combining transcription factor overexpression with extracellular signaling cues (Bar-Nur et al., 2014). We conducted a systematic screen for pro-myogenic factors by modulating key signaling cascades that are implicated in primitive streak formation, somitogenesis, and myogenesis (Figure S3A). We found that the addition of all-*trans* retinoic acid (RA) in conjunction with MYOD1 overexpression was sufficient for rapid and deterministic conversion of hPSCs into myogenin and myosin heavy chain double-positive myocytes after 5 days of induction (Figures 3C and S3A). The effect of RA was concentration dependent (data not shown), and mediated at least in part through the receptor isoforms RARα and RARβ (Figures S3B and S3C).

Following minor optimization of the culture conditions (see the Supplemental Experimental Procedures), we arrived at a protocol resulting in nearly pure induced skeletal myocytes (i-Myocytes). Reprogrammed cells developed typical spindle-like, elongated morphology, underwent extensive cell fusion, and exhibited strong and homogeneous myogenic marker expression on mRNA and protein levels (Figures 3D–3H, S3D, and S3E; Movie S2). Furthermore, the addition of nanomolar concentrations of acetylcholine (ACh) or the selective ACh-receptor agonist carbachol resulted in muscle fiber contraction, demonstrating the functionality of the i-Myocytes (Movie S3). Similar results were obtained with i-Myocytes generated from MYOD1 OPTi-OX hiPSCs (Figure S3F). Importantly, induction efficiency did not decrease over extended culture periods (>50 passages, Figure 3H), thus demonstrating the robustness and reproducibility of this method. Finally, we noted that the levels of the MYOD1 transgene following induction positively correlated with conversion efficiency (Figures 3I and 3J), which highlights the importance of a robust gene-delivery method. In conclusion, these data demonstrated that the OPTi-OX platform enables robust and rapid forward programming of hPSCs into skeletal myocytes.

### Human Induced Oligodendrocytes

Encouraged by our results deriving neurons and myocytes, we sought to utilize the same overexpression system to develop a forward programming protocol for oligodendrocytes. Oligodendrocytes are of critical importance for CNS function and their loss or dysfunction plays a key role in many neurological diseases. Unlike neurons (Zhang et al., 2013), protocols for efficient generation of human oligodendrocytes from renewable sources remain an unmet need: currently available hPSC differentiation protocols are extremely long (up to 200 days) and yield heterogeneous cell populations (Douvaras et al., 2014, Stacpoole et al., 2013, Wang et al., 2013).

**Figure 4:**
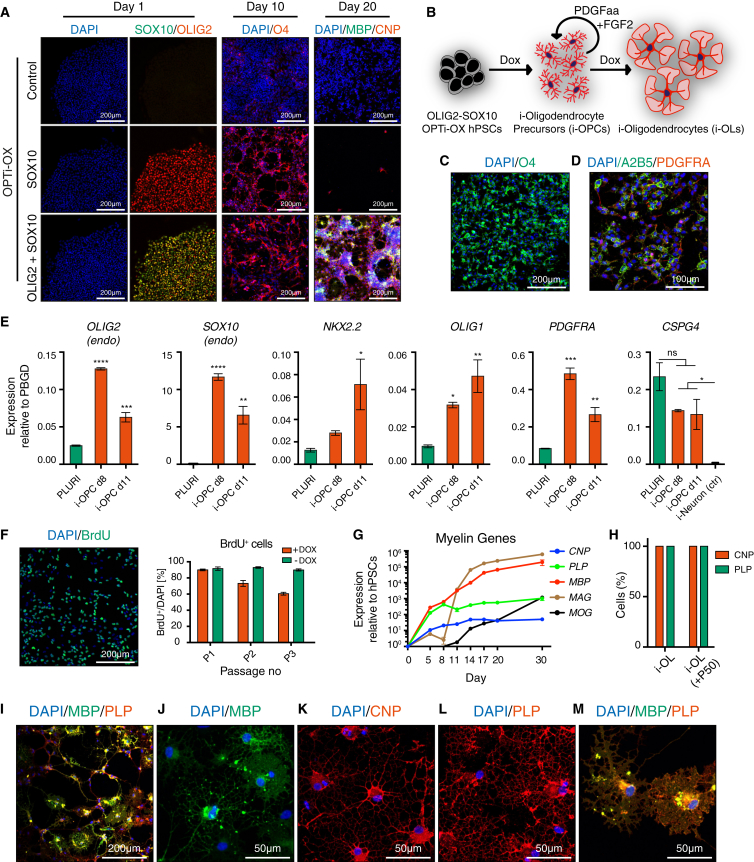
Forward Programming of hPSCs into Oligodendrocytes (A) ICC for inducible transgenes after 1 day of induction (left column), the OPC marker O4 after 10 days (middle), and the oligodendrocyte markers CNP and MBP after 20 days (right). (B) Experimental approach for rapid conversion of OLIG2-SOX10 OPTi-OX hPSCs into the oligodendrocyte lineage cells (i-OPCs and i-OLs). (C and D) Characterization of i-OPCs by ICC for OPC surface markers (A2B5, O4, PDGFRA). (E) Characterization of i-OPCs by qPCR compared with hPSCs (PLURI). As transcription of *CSPG4* (NG2) was also detected in hPSCs, we included i-Neurons as negative control (n = 3 biological replicates; mean ± SEM; all values relative to *PBGD*; one-way ANOVA with post hoc Dunnett's test; ^∗^p < 0.05; ^∗∗^p < 0.01, ^∗∗∗^p < 0.001, ^∗∗∗∗^p < 0.0001; ns, p > 0.05). (F) Immunostaining for BrdU (left panel) and quantification of BrdU-positive cells following three serial passages of i-OPCs every 4 days and concomitant BrdU-pulses (n = 3 biological replicates; mean ± SEM; P, passage). (G) qPCR of the temporal expression pattern of genes encoding for the myelin-associated proteins (*CNP*, *MAG*, *MBP*, *MOG*, and *PLP*) during i-OL generation. OLIG2-SOX10 OPTi-OX hPSCs were induced in oligodendrocyte medium supplemented with PDGFaa and FGF2. After 1 week of induction, mitogens were withdrawn to enable terminal differentiation (n = 3 biological replicates; mean ± SEM; all values relative to *PBGD* and normalized to pluripotency). (H) Quantification of CNP and PLP expressing i-OLs derived from OLIG2-SOX10 OPTi-OX hPSCs after 20 days of induction by ICC. Undifferentiated cells were used as negative control, and figures are reported for newly isolated OLIG2-SOX10 OPTi-OX hPSCs and after 50 passages (+P50). (I–M) ICC providing an overview (I) and high-magnifications (J–M) of mature pre-myelinating oligodendrocytes.

We generated OPTi-OX hPSCs bearing inducible SOX10 either alone or in combination with OLIG2 in the form of a polycistronic expression cassette (Figure 4A). Although cells induced with SOX10 alone robustly expressed the oligodendrocyte precursor (OPC) marker O4 after 10 days of induction, these cells failed to differentiate into myelin-expressing cells and died (Figure 4A). In contrast, the OLIG2-SOX10 overexpressing cells progressed from an O4-positive progenitor stage into a mature CNP/MBP-positive phenotype after 20 days of induction (Figure 4A). Moreover, gene expression analysis confirmed that OLIG2-SOX10 OPTi-OX hPSCs induced in oligodendrocyte medium (Douvaras et al., 2014) supplemented with the mitogens PDGFaa and FGF2 first passed through an OPC-like stage, during which they remained proliferative and co-expressed typical OPC markers (Figures 4B–4F). Remarkably, following withdrawal of mitogens, i-OPCs differentiated into mature oligodendrocytes, expressing the typical myelin-associated proteins (Figures 4G–4M).

## Discussion

OPTi-OX is the result of a systematic effort to optimize gene expression in hPSCs. It relies on a dual GSH-targeting strategy for the Tet-ON system, overcoming the limitations of viral-mediated transgene delivery forward programming protocols (Abujarour et al., 2014, Darabi et al., 2012, Zhang et al., 2013) and allows stronger and more controlled transgene overexpression compared with previous targeting approaches (González et al., 2014, Hockemeyer et al., 2009, Ordovás et al., 2015). Table S2 compares the gene-delivery methods that have been used for transcription factor expression in different hPSC forward programming approaches. Moreover, site-specific insertion of the two components of the inducible gene expression system minimizes genomic off-target effects and together with the chemically defined medium compositions enhances the reproducibility of the protocols.

The functionality of our platform is exemplified through the production of several cell types. First, we show that NGN2 and MYOD1 OPTi-OX hPSCs can be used as an inexhaustible source for highly scalable, rapid, single-step, virus-free, chemically defined, fully reproducible, and deterministic generation of i-Neurons and i-Myocytes.

Finally, we successfully applied the OPTi-OX platform to develop a forward programming protocol for generating human oligodendrocytes. Recent studies demonstrated that forced expression of transcription factors allows direct conversion of rodent fibroblasts (Najm et al., 2013, Yang et al., 2013), and primary human fetal neural stem cells (Wang et al., 2014) into OPCs, but the reprogramming of renewable human cell sources into oligodendrocytes has not been reported. While i-OPCs undergo the expected morphological changes, and express mature markers in monocultures in vitro, further characterization of the cells using co-culture models and transplantation into myelin-deficient mutants is needed.

Human oligodendrocytes are of considerable interest for several applications. The efficiency and speed of the presented forward programming system will enable high-throughput drug screens and toxicology testing, in vitro modeling of hereditary leukodystrophies, and the development of cell-transplantation strategies (Goldman et al., 2012).

Transcription factor combinations for direct cellular reprogramming into many cell types of clinical interest are now available, including cardiomyocytes (Ieda et al., 2010), pancreatic β cells (Zhou et al., 2008), and hepatocytes (Huang et al., 2014). We anticipate that the OPTi-OX platform will be applicable for the generation of many other cell types. Overall, the presented method can provide the basis for inexhaustible, high-throughput, homogeneous, and large-scale manufacturing of many human cell types.

## Experimental Procedures

### Gene Targeting

Targeting of the hROSA26 and AAVS1 locus was performed as described recently (Bertero et al., 2016). Targeting of the hROSA26 locus was done by nucleofection. Neomycin-resistant colonies were picked and screened by genotyping. Correctly hROSA26-rtTA-targeted clones were subsequently targeted with the inducible transgene cassette in the AAVS1 locus by lipofection. Resulting puromycin-resistant colonies were picked and re-analyzed by genotyping.

### Inducible Transgene Expression and Forward Programming

Inducible overexpression was performed with dual GSH-targeted OPTi-OX hPSCs. Expression of inducible transgenes was prompted by adding dox to the culture medium. For forward programming into neurons, skeletal myocytes, and oligodendrocytes, standard medium conditions for the derivation of the respective cell types were used. Gene and protein expression analysis was performed as described recently (Bertero et al., 2016). Please refer to the Supplemental Experimental Procedures for details on culture conditions and analysis techniques.

## Author Contributions

M.P. conceived the study, designed and performed experiments, analyzed data, and wrote the first draft of the manuscript. D.O. designed and performed experiments and analyzed the data. A.B. designed and performed experiments, analyzed data, and wrote the manuscript. J.M.T. performed additional experiments. R.A.P. provided expert advice. L.V. supervised and supported the study. M.R.N.K. conceived, supervised, supported the study, and finalized the manuscript.

## Supplemental Information

Document S1. Supplemental Experimental Procedures, Figures S1–S3, and Tables S1 and S2

Movie S1. Time-Lapse of Neuron Induction, Related to Figure 2

Movie S2. Time-Lapse of Myocyte Induction, Related to Figure 3

Movie S3. Response of Induced Myocytes to ACh-Receptor Stimulation, Related to Figure 3

Document S2. Article plus Supplemental Information
