# Supplementary material for: Inducible and Deterministic Forward Programming of Human Pluripotent Stem Cells into Neurons, Skeletal Myocytes, and Oligodendrocytes
Source: Stem Cell Reports. 2017 Mar 23;8(4):803–12. doi: 10.1016/j.stemcr.2017.02.016 (PMC5390118; doi:10.1016/j.stemcr.2017.02.016)
Supplement: Document S1. Supplemental Experimental Procedures, Figures S1–S3, and Tables S1 and S2 [file mmc1.pdf]

Stem Cell Reports, Volume *8*

Supplemental Information

Inducible and Deterministic Forward Programming of Human Pluripotent Stem Cells into Neurons, Skeletal Myocytes, and Oligodendrocytes

Matthias Pawlowski, Daniel Ortmann, Alessandro Bertero, Joana M. Tavares, Roger A. Pedersen, Ludovic Vallier, and Mark R.N. Kotter

Supplemental Figures

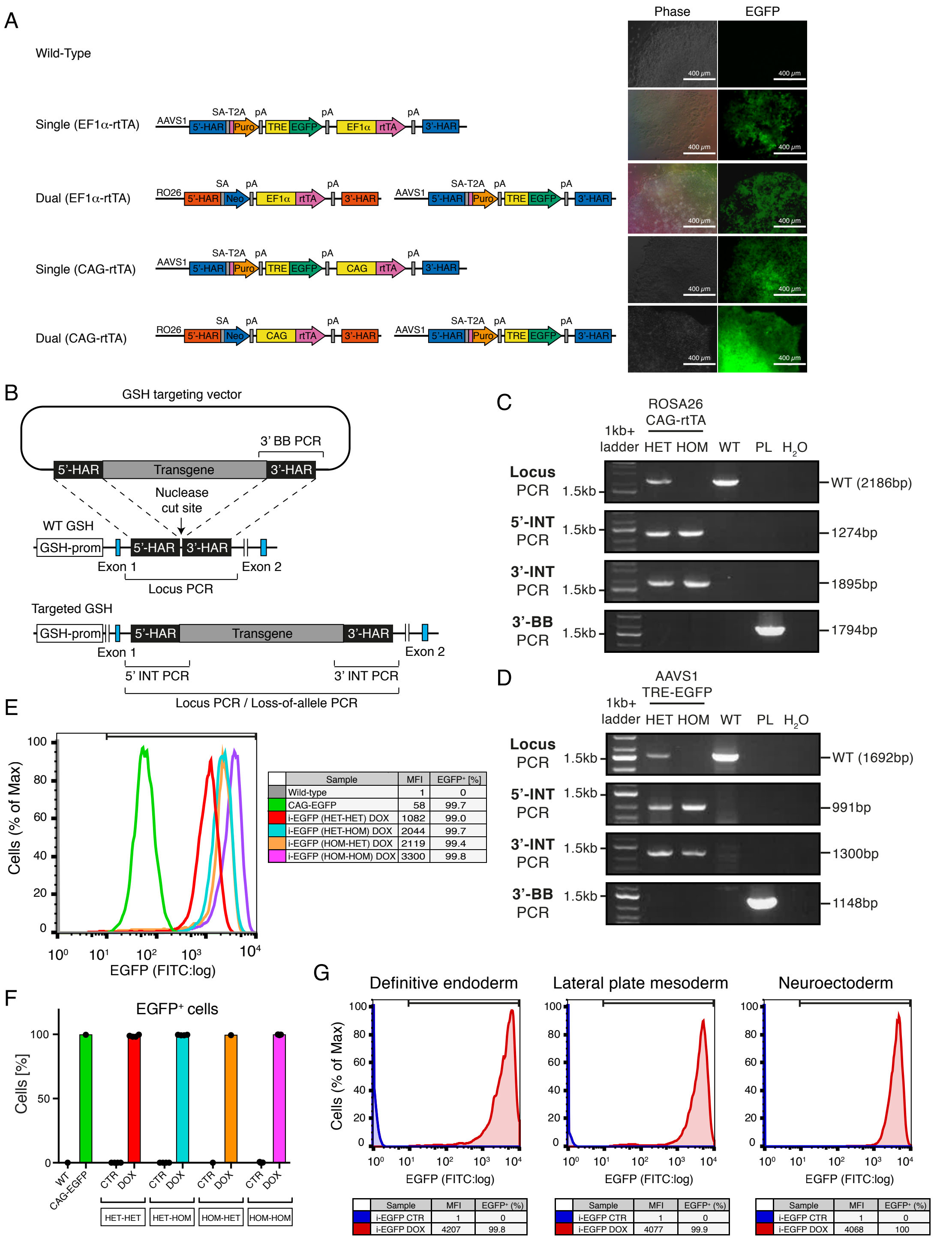

Figure S1. Related to Figure 1: Generation of hPSCs with inducible EGFP expression

(A) Schematic of the four different GSH (hROSA26 and AAVS1) targeting strategies: the two components (rtTA trans-activator cassette and inducible TRE-EGFP responder cassette) of the Tet-ON system were targeted either separately into the hROSA26 and AAVS1 locus, respectively, or together into the same allele of the AAVS1 locus by using an all-in-one donor vector design. For both configurations rtTA expression is controlled either by an EF1α- or CAG-promoter. The resulting cell lines were treated with dox for 5 days. Phase contrast and EGFP-images demonstrate homogeneous EGFP expression only in the dual GSH-targeting strategy in which rtTA expression is under control of the CAG promoter. Abbreviations: *RO26*: human ROSA26 (hROSA26) locus; *AAVS1*: AAVS1 locus; *5'-HAR/3'-HAR*: upstream/downstream homology arm. *SA*: splice acceptor; *T2A*: T2A peptide

(ribosomal skipping signal); *Neo*: neomycin resistance gene (neomycin phosphotransferase II); *Puro*: puromycin resistance gene (puromycin *N*-acetyltransferase); *pA*: polyadenylation signal; *EF1α*: human elongation factor 1α promoter; *CAG*: CMV early enhancer, chicken β-actin and rabbit β-globin hybrid promoter; *EGFP*: enhanced green fluorescent protein; *CAG*: **(B)** Schematic of the genotyping strategy used to identify correctly targeted hROSA26 and AAVS1 targeted hPSC lines. *GSH-prom*: hROSA26 and AAVS1 promotor, respectively; *WT*: wild-type; *Transgene*: entire exogenous sequence integrated following targeting. *Locus PCR*: PCR spanning the targeted locus with both primers binding exclusively to genomic DNA outside the genomic sequence corresponding to the homology arms. Note that due to its high GC-content the CAG promoter cannot be amplified by routine PCR. Therefore, correct insertion of the CAG-containing expression cassette results in loss of a PCR amplicon. The presence of the wild-type band indicates the presence of non-targeted alleles, and loss of the wild-type band indicates homozygous targeting. *5'-INT/3'-INT PCRs*: PCRs spanning the 5'- and 3'-insertion site, respectively. Correctly sized PCR amplicons indicate correct integration. *3'-BB PCR*: PCR spanning the homology arm/targeting vector backbone junction. The presence of a PCR product indicates non-specific off-target integration of the donor plasmid. **(C-D)** Genotyping results for selected hROSA26-CAG-rtTA targeted (C) and AAVS1-TRE-EGFP re-targeted hPSCs (D). Heterozygous (HET) and homozygous (HOM) lines for each allele are shown. *1kb+*: 1kb plus DNA ladder; *WT*: wild-type hPSCs; *PL*: targeting plasmid; *H_2O*: water control. Refer to Supplemental Table 1 for a summary of all genotyping results and Supplemental Table 2 for the sequence of all genotyping primers. **(E)** Representative flow cytometry analysis of the various dual GSH-targeted inducible EGFP hPSCs described in figure 1B. Cells were analyzed in control conditions (no dox, CTR) or following 5 days of dox treatment (DOX). An hESC line in which EGFP expression is under control of a CAG promoter in the AAVS1 locus (Bertero et al. 2016) is included as a positive control and for comparison. Note that the FITC (EGFP) acquisition settings were set to enable plotting of the highest levels of EGFP expression. Thus, the negative control (wild-type) is located directly adjacent to the left y-axis. **(F)** Percentage of EGFP positive cells in the various dual GSH-targeted inducible EGFP hESCs (each data point represents an individual clonal line; n=1-5) **(G)** Representative EGFP flow cytometry plots in EGFP OPTi-OX hPSCs that were differentiated into the three germ layers. EGFP was induced with dox for the duration of the differentiation protocol (3 to 6 days, refer to Supplemental Methods).

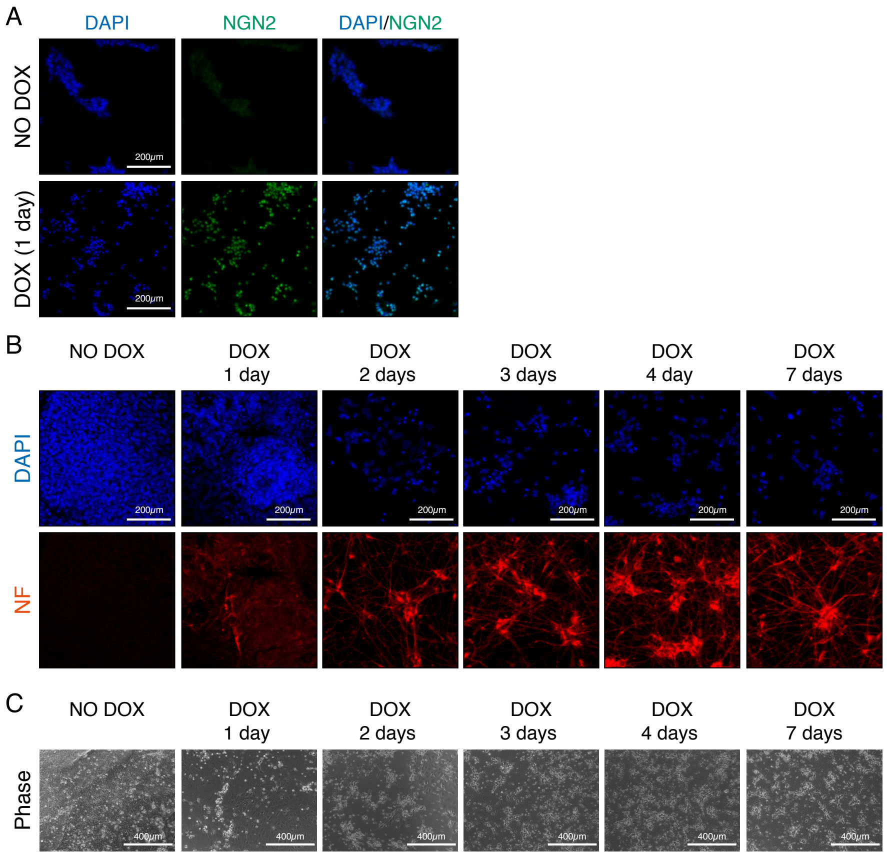

Figure S2. Related to Figure 2: Characterization of induced neurons

(A) Representative immunocytochemistry for NGN2 in NGN2 OPTi-OX hPSCs before (NO DOX) and 24h after induction with dox (DOX). The staining demonstrates robust and homogeneous induction of transgene expression. **(B)** Representative immunocytochemistry for the pan-neuronal marker neurofilament (NF) 7 days after the start of induction with dox. NGN2 OPTi-OX hPSCs were treated with doxycycline for 0, 1, 2, 3, 4 or 7 days, respectively. A minimum of 4 days of dox treatment was sufficient to yield the same number of neurons compared to the addition of dox supplementation throughout the experiment. **(C)** Representative phase contrast images of the same cultures shown in panel B.

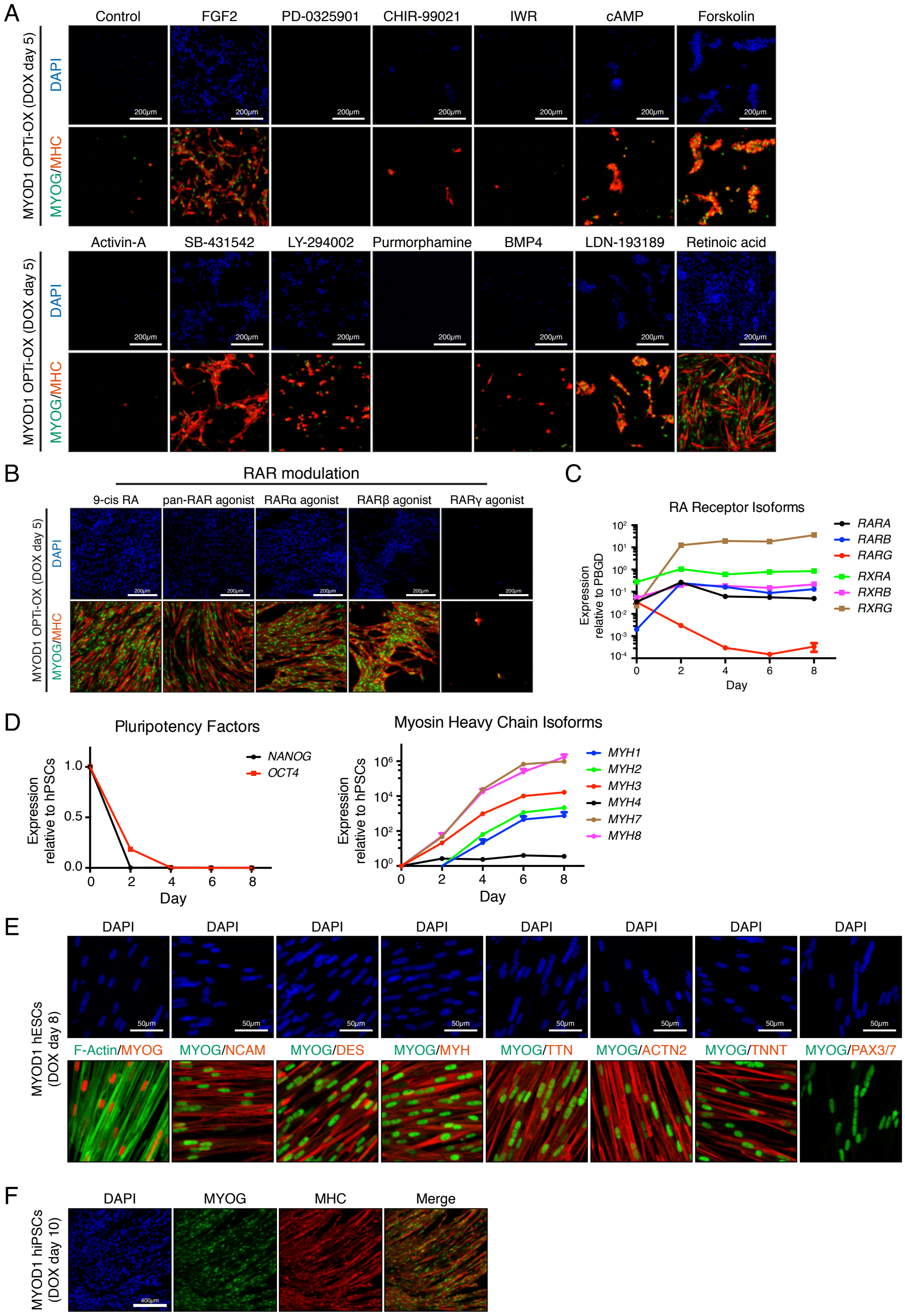

Figure S3. Related to Figure 3: Screening for chemically-defined culture conditions for the induction of skeletal myocytes and characterization of skeletal myocytes

(A) Screening for pro-myogenic factors: MYOD1 overexpression was initiated in DMEM basal media supplemented with different modulators of signaling cascades implicated in mesoderm patterning, somitogenesis and myogenesis. Shown is the immunocytochemistry for the myocyte markers myogenin (MYOG) and myosin heavy chains (MYH) 5 days post induction. This screen suggested that FGF2, forskolin (an adenylyl cyclase activator), and SB-431542 (an Activin/Nodal signaling inhibitor) have minor beneficial effects on cell survival and induction of myogenin and myosin heavy chain. However, the addition of all-trans retinoic acid (RA) led to the best outcome regarding survival, cell morphology and marker expression. This culture condition was chosen for the generation of induced myocytes (i-Myocytes) from MYOD1 OPTi-OX hPSCs. **(B)** RA was replaced with a range of selective, isoform-specific RA-Receptor agonists throughout the myocyte induction protocol (pan-RAR/RXR agonist: 9-cis RA 1μM; pan-RAR agonist: TTNPB 1μM; RARα agonist: BMS753 1μM; RARβ agonist: CD2314 1μM; RARγ agonist: BMS961 1μM). Immunocytochemistry for myogenin and myosin heavy chain 5 days post induction demonstrated that the enhancing effect of RA during myocyte induction are mediated through the RA receptor isoforms RARα and RARβ, but not RARγ. **(C)** qPCR analysis of the six retinoid and rexinoid receptors during myocyte induction demonstrated expression of RARα, RARβ and all three RXR isoforms, but not of RARγ throughout the course of i-Myocyte generation, consistent with the expression pattern of RA receptors during developmental myogenesis (n=3 biological replicates; mean ± SEM). **(D)** qPCR analysis of pluripotency factors and myosin heavy chain (MHC) isoforms during myocyte induction demonstrated rapid downregulation of the pluripotency factors *NANOG* and *OCT4* (left panel), and strong upregulation of all major human skeletal myocyte-specific MHC isoforms (encoded by the *MYH* gene family) during i-Myocyte generation. The upregulated MHC-isoforms included the two isoforms that are expressed during embryonic and postnatal muscle development (embryonic isoform MYH3; neonatal isoform MYH8), and three isoforms that are expressed in adult human skeletal muscle [MYH7 in slow-twitching (type I) fibers; MYH2 in fast-twitching fatigue-resistant (type IIa) fibers, and MYH1 in fast-fatigable (type IIx) fibers]. In contrast, MYH4 which represents the constituting MHC-isoform in fast-twitching, fast-fatigable myocyte fibers in cats is not expressed in significant amounts in humans (<1%) and is also not induced throughout the forward programming time course (n=3 biological replicates; mean ± SEM). **(E)** i-Myocytes express a broad range of typical marker proteins, including F-actin (visualized through AlexaFluor488-conjugated Phalloidin toxin), neural cell adhesion molecule (*NCAM*), desmin (*DES*), myosin heavy chains (*MYH*), titin (*TTN*), α-actinin (*ACTN2*) and troponin T (*TNNT*), but not the myoblast progenitor markers *PAX3* and *PAX7*. All samples were co-stained with myogenin. **(F)** Human MYOD1 OPTi-OX hiPSCs underwent myogenic induction comparably to their hESC counterparts, as demonstrated by immunocytochemistry for myogenin and myosin heavy chain 10 days post induction.

Supplemental Tables

Table S1. Summary of genotyping results

Locus	Cell-Line	Trans-gene	# clones picked (a)	# clones no on-tar. Integrat. (b)	# clones HET + off-targ. (c)	# clones HOM +off-targ. (c)	# clones HET (d)	# clones HOM (d)	Efficiency no off-tar. [%] (e)	Efficiency total [%] (f)
ROSA26	H9	rtTA	23/27/60*	2/3/1*	7/13/36*	5/3/6*	8/8/14*	1/0/3*	39/30/28*	91/89/98*
ROSA26	iPSC	rtTA	48	8	11	2	25	2	56	83
AAVS1	H9	EGFP	12/12/24*	2/1/2*	0/0/0*	4/5/11*	0/1/4*	6/5/7*	50/50/46*	83/92/92*
AAVS1	H9	NGN2	6	0	0	0	0	6	100	100
AAVS1	iPSC	NGN2	3	0	0	2	1	0	33	100
AAVS1	H9	MYOD1	12	2	0	3	0	7	58	75
AAVS1	iPSC	MYOD1	3	0	1	1	0	1	33	100

Refer to Figure S1B for a schematic and additional explanations of the PCR genotyping strategy used.
(a) Number of clones that were picked for expansion and genotyping following nucleofection (for hROSA26 targeting) and lipofection (for AAVS1 targeting), respectively. The total numbers of drug resistant colonies (from which the stated numbers of colonies were picked) were approximately 200 colonies per 10cm dish following nucleofection, and 20 colonies per well of 6-well plate following lipofection.
(b) Incorrect targeting: No evidence of targeting (lack of bands in 5'- and 3'-integration PCR and presence of WT band in locus PCR) or evidence of targeting, but incorrect size of 5'- or 3'-integration PCR.
(c) Correct on-target integration with additional random integration of the plasmid (bands in 3'-backbone PCR).
(d) Correct on-target integration (HET, heterozygous; HOM, homozygous).
(e) Percentage of clones with correct on-target integration (without additional off-target integration).
(f) Percentage of clones with correct on-target integration (with or without additional off-target integration).
* The three numbers are from three different targeting experiments in hESCs.

Table S2. Overview of gene delivery strategies in forward programming experiments

Gene Delivery	Off-switch (a)	Resistance to Silencing (b)	Expression level (c)	Genomic homogeneity /integrity of targeted cells (d)	Independency of optimized viral transduction (e)	Repro-ducibility (f)	Reference
*acute Lenti PGK	0	+	+	0	0	+	Albini et al. 2013
*acute Lenti EF1α	0	+	+	0	0	+	Moreau et al. 2016
*acute Lenti i-TRE	+	+	0	0	0	+	Pang et al. 2011
*acute Lenti i-TRE (select)	+	++	++	0	+	++	Zhang et al. 2013
**clonal Lenti/Pig i-TRE	+	++	+	+	++	0	Abujarour et al. 2014 Tanaka et al. 2013
Single GSH i-TRE	++	++	++	+++	+++	+++	This study
Dual GSH i-TRE	+++	+++	+++	+++	+++	+++	This study

*acute: hPSCs are transduced with lentiviral vectors (Lenti) - encoding either a constitutive or inducible reprogramming factor expression cassette - at the beginning of each individual reprogramming experiment.
**clonal: hPSCs are transduced with lentiviral vectors (Lenti) or piggyback vectors (Pig) – encoding an inducible reprogramming factor cassette – clonally expanded, and each clonal line is subsequently analyzed for its transgene expression and forward programming capacity.

(a) An optimal gene delivery system for forward programming contains an on-/off-switch to allow for controlled transcription factor expression during the reprogramming process and turning-off after a full cellular conversion has been achieved. This cannot be achieved by using constitutive promoters for transcription factor expression such as the PGK- or EF1α-promoter. Moreover, it has been demonstrated that significant leaky expression may occur following random genomic integration of the inducible (i) TRE-promoter or by placing the i-TRE in close vicinity to a strong constitutive promoter.
(b) An optimal gene delivery system for forward programming renders the reprogramming factors immune to endogenous silencing mechanisms. Acute viral transduction of hPSCs invariably leads to transgene silencing in a large proportion of transfected cells. This proportion can be reduced by including an antibiotic resistance gene to allow for positive selection of successfully transduced cells. However, the expression levels of these genes required for antibiotic resistance are typically orders of magnitudes smaller than expression levels of reprogramming factors required for the initiation of a successful conversion process. Therefore, in this case, positive selection does not accurately predict successful conversion.
(c) An optimal gene delivery system for forward programming enables very high transgene expression levels to facilitate a successful conversion process (see Fig. 3I-J). Transgene expression levels are a direct function of the strength of the promoter and the effects of silencing/variegation mechanisms, which occur in an integration site-dependent manner.
(d) An optimal gene delivery system for forward programming ensures that all successfully reprogrammed cells are genetically identical and free of genomic alterations that potentially interfere with target cell function.
(e) An optimal gene delivery system for forward programming does not rely on optimization of protocols for the production and titration of lentiviral vectors and the subsequent transduction of hPSCs, which represent a traditionally difficult to transduce cell type.
(f) An optimal gene delivery system for forward programming allows full predictability and reproducibility of the resulting forward programming protocol among different labs and users. Naturally, this is impossible to achieve if the protocol relies on the selection of a clonally expanded line with a randomly integrated transgene. Moreover, it is also difficult to achieve when hPSC-transduction protocols with highly-concentrated lentiviral vectors are required.

Supplemental Movies

Movie S1. Related to Figure 2. Time-lapse of neuron induction

Movie S2. Related to Figure 3. Time-lapse of myocyte induction

Movie S3. Related to Figure 3. Response of induced myocytes to ACh-Receptor stimulation

Supplemental Experimental Procedures

hPSC maintenance culture and germ layer differentiation

Feeder- and serum-free hESC (H9 line; WiCell) and hiPSC (Cheung et al. 2012) culture was performed as previously described (Vallier 2011). Briefly, cells were plated on gelatin/MEF media-coated culture dishes [MEF-media consists of Advanced DMEM/F12 (90%, Gibco), fetal bovine serum (10%, Gibco), L-Glutamine (1mM, Gibco), 2-Mercaptoethanol (0.1mM, Sigma-Aldrich) and Penicillin/Streptomycin (1%, Gibco)], and cultured in chemically defined media [CDM, consisting of IMDM (50%, Gibco), F12 (50%, Gibco), concentrated lipids (100x, Gibco), monothioglycerol (450µM, Sigma-Aldrich), insulin (7µg/ml, Roche), transferrin (15µg/ml, Roche), bovine serum albumin fraction V (5mg/ml, Europa Bioproducts), and Penicillin/Streptomycin (1%)] supplemented with Activin-A (10ng/ml) and FGF2 (12ng/ml; both from Marko Yvonen, Department of Biochemistry, University of Cambridge). Cells were passaged in small clumps using collagenase every 5-6 days.

Differentiation of hPSCs into the germ layers was induced in adherent hESC cultures in accordance to previously published directed differentiation protocols. Briefly, definitive endoderm was derived by culturing hPSCs for 3 days in CDM-PVA (without insulin) supplemented with FGF2 (20ng/ml), Activin-A (100ng/ml), BMP4 (10ng/ml, Marko Hyvonen, Dept. of Biochemistry, University of Cambridge), and LY-294002 (10µM, Promega) (Touboul et al. 2010). For derivation of neuroectoderm, hPSCs were cultured for 6 days in CDM-BSA supplemented with SB-431542 (10µM, Tocris), LDN-193189 (0.1µM, Tocris) and RA (0.1µM, Sigma) (Douvaras et al. 2014). Lateral plate mesoderm was obtained by culturing hPSCs for 36h in CDM-PVA supplemented with FGF2 20ng/ml, BMP4 (10ng/ml; R&D), and LY294002 (10µM), and for 3.5 subsequent days in CDM-PVA supplemented with FGF2 (20ng/ml) and BMP4 (50ng/ml) (Cheung et al. 2012).

Gene targeting constructs and molecular cloning

We recently described the design and construction of the hROSA26 gRNA/Cas9n expression plasmids and of the hROSA26 targeting vector, and demonstrated that this targeting strategy allows for homogeneous, strong and stable expression of targeted transgenes in hPSCs and their differentiated progenies (Bertero et al. 2016). The pR26_CAG-rtTA targeting vector was constructed by cloning the coding sequence of a third generation rtTA (Zhou et al. 2006) (PCR-amplified from pLVX-Tet3G) into the BamHI/MluI sites of pR26_CAG-EGFP (Bertero et al. 2016) thus replacing the EGFP sequence.

AAVS1 ZFN expression plasmids were a generous gift of Dr. Kosuke Yusa, Wellcome-Trust Sanger Institute (Bertero et al. 2016). The inducible EGFP AAVS1 targeting vector was constructed by Gibson Assembly (New England Biolabs) in which three inserts were ligated into the EcoRI/HindIII sites of the multiple cloning site of the pUC19 vector (Thermo Fisher Scientific): The first insert comprised the upstream AAVS1 homology arm, a splice acceptor, a T2A-site and the puromycin resistance cassette (all these elements were PCR-amplified from pTRE-EGFP; addgene 22074, deposited by Rudolf Jaenisch). The second insert contained the inducible TRE3G promoter (PCR-amplified from pLVX-TRE3G). The third insert comprised the EGFP expression cassette and the AAVS1 downstream homology arm (PCR-amplified from pTRE-EGFP; addgene 22074, deposited by Rudolf Jaenisch). The resulting plasmid was termed pAAV_TRE-EGFP. The pAAV_TRE-NGN2 and pAAV_TRE-MYOD1 targeting vectors were constructed by cloning the NGN2 and MYOD1 coding sequence, respectively (NGN2: PCR-amplified from pLVX-TRE-NGN2 (Ladewig et al. 2012), gift from Oliver Brüstle; MYOD1, OLIG2, SOX10: PCR-amplified from commercially available cDNA plasmids, Open Biosystems) into the SpeI/EcoRI sites of pAAV_TRE-EGFP, thus replacing the EGFP sequence.

Gene targeting

Targeting of the hROSA26 locus (Irion et al. 2007) was performed by nucleofection. Human PSCs were dissociated to single cells with TrypLE Select (Gibco), and $2x10^6$ cells were nucleofected (100µl reaction volume; total of 12µg of DNA, which was equally divided between the two gRNA/Cas9n plasmids and the targeting vector) using the Lonza P3 Primary Cell 4D-Nucleofector X Kit and cycle CA-137 of the Lonza 4D-Nucleofector System. Nucleofected hPSCs were plated onto irradiated multi-drug resistant (DR4) mouse embryonic fibroblasts and cultured in KSR media [consisting of Advanced DMEM/F12 (80%), knock-out serum replacer (20%, Gibco), L-Glutamine (1mM), 2-Mercaptoethanol (0.1mM) and Penicillin/Streptomycin (1%)] supplemented with FGF2 (4ng/ml). Y-27632 (5µM, Tocris) was added for 24h before and after nucleofection to promote cell survival. After 3-6 days, neomycin-resistant hPSCs were selected by adding G418 (50 µg/ml, Sigma-Aldrich) for 7-10 days.

Subsequently, individual clones were picked, expanded in feeder-free conditions and finally analyzed by genotyping.

Targeting of the AAVS1 locus (Hockemeyer et al. 2009) was performed by lipofection. Human PSCs were seeded in feeder-free conditions in 6-well plates, and transfected 48h after passaging. Transfection was performed in Opti-MEM (Gibco) supplemented with Lipofectamine2000 (10 µl/well, Thermo Fisher Scientific) and a total of 4 µg of DNA (equally divided between the two AAVS1 ZFN plasmids and the targeting vector) for 24h. After 3-5 days, resistant hPSCs were selected by adding puromycin (1µg/ml, Sigma-Aldrich) for 5-8 days. Subsequently, individual clones were picked, expanded and analyzed by genotyping.

Drug-resistant hPSC clones from targeting experiments were screened by genomic PCR to verify site-specific transgene integration, to determine the number of targeted alleles, and to exclude off-target integrations. PCRs were performed with LongAmp Taq DNA Polymerase (New England Biolabs). The primer combinations used for the various targeting vectors are reported in supplemental experimental procedures. The results of all targeting experiments are summarized in Supplemental Table 1. Karyotype analysis was performed by standard G banding techniques (Medical Genetics Service, Cambridge University Hospitals). To prepare the targeted human PSCs for chromosome analysis, cells were incubated in fresh culture media supplemented with Y-27632 (5µM, Tocris) and KaryoMAX Colcemid (100ng/ml, Gibco) for 4h at +37°C. Subsequently, cells were harvested as single cells, washed, and pelleted. Nuclei swelling and spreading of the chromosomes was achieved by treatment with hypotonic 0.055 M KCl-solution for 5 minutes. Finally, cells were fixed and preserved in methanol and glacial acetic acid (ratio 3:1).

Induction of neurons
Pluripotent NGN2 OPTi-OX cells were dissociated into single cells with TrypLE and plated onto Matrigel (35 µg/cm^2, Scientific Laboratory Supplies) coated dishes at a density of 75.000 cells per well of a 12-well plate. Forward programming was initiated 24 hours after the split. The induction was performed in DMEM/F12 (Gibco) supplemented with Glutamax (100x, Gibco), Non-Essential Amino Acids (100x, Gibco), 2-Mercaptoethanol (50µM), Penicillin/Streptomycin (1%), and dox (1µg/ml). After 2 days of induction, the medium was switched to Neurobasal-medium supplemented with Glutamax (100x), B27 (50x, Gibco), BDNF (10 ng/ml, Peprotech), NT3 (10 ng/ml, R&D Systems), Penicillin/Streptomycin (1%), and dox (1µg/ml). These culture conditions were based on previously described chemically-defined neuronal basal culture media (Zhang et al. 2013), and used for all experiments presented. Cells were cultured for 7-14 days before analysis.

Induction of skeletal myocytes
Pluripotent OPTi-MYOD1 cells were dissociated into single cells with TrypLE and plated onto gelatine/MEF-medium coated dishes at a density of 100.000 cells per well of a 12-well plate. Forward programming was initiated 24 hours after the split. Unless stated otherwise, the induction was performed in DMEM (Sigma-Aldrich) supplemented with L-Glutamine (2mM), 2-Mercaptoethanol (50µM), Penicillin/Streptomycin (1%), insulin (7µg/ml), all-trans retinoic acid (1µM, Sigma-Aldrich), and doxycycline (1µg/ml). After 5 days of induction, the medium was supplemented with CHIR99021 (3µM, Tocris) and heat-inactivated horse serum (2%, Gibco) to enhance maturation of the induced myocytes as previously described (Abujarour et al. 2014). Unless stated otherwise, these culture conditions were used in all experiments described in this paper. Additional reagents were tested during protocol optimization (see Fig. S2): DMEM/F12 (Gibco), IMDM (Gibco), DMEM (Sigma), α-MEM (Sigma), BMP4 (10ng/ml, Marko Hyvonen, Dept. of Biochemistry, University of Cambridge), cAMP (1µM, Sigma), Forskolin (20ng/µl, Tocris), IWR-1 (1µM, Tocris), LY-294002 (10µM), Purmophamine (1µM, Tocris), PD-0325901 (1µM, Tocris), LDN-193189 (0.1µM, Tocris), SB-431542 (10µM, Tocris), 9-cis RA (1µM, Sigma) TTNPB (1µM, Tocris), BMS753 (1µM, Tocris), CD2314 (1µM, Tocris), BMS961 (1µM, Tocris).

Induction of oligodendrocytes
Pluripotent OLIG2-2A-SOX10 OPTi-OX hPSCs were grown in colonies on gelatine/MEF coated culture dishes. Before the start of induction, they were treated with SB and LDN overnight. The following day induction was initiated in CDM supplemented with dox (1µg/ml) and RA (0.1 µM). One day after induction, cells were split in CDM supplemented with RA (0.1 µM), PM (1µM), and Y-27632 (5µM), PDGFaa (20ng/ml, Peprotech), FGF2 (5ng/ml) onto PDL/laminin coated culture dishes (100.000 cells per well of a 12 well-plate). The following day cells were switched to oligodendrocyte

media consisting of DMEM/F12, supplemented with Glutamax (100x), Non-Essential Aminoacids (100x), 2-Mercaptoethanol (1000x), Penicillin-Streptomycin (100x), N2 Supplement (100x), B27 Supplement (50x), Insulin 7μg/ml (Marko Hyvonnen), T3 60ng/ml (Sigma), Biotin 100ng/ml (Sigma), and db-cAMP 1μM (Sigma). Oligodendrocyte medium was supplemented with dox (1μg/ml), PDGFaa (20 ng/ml), FGF2 (5 ng/ml), RA (0.1 μM) and PM (1 μM). Seven days post induction RA and PM was withdrawn. To keep induced cells in a proliferative state, cells were passaged every 4 days (75.000 cells per well of a 24-well plate) in the continued presence of the mitogens PDGFaa and FGF2. For differentiation of proliferative oligodendrocyte precursors, PDGFaa and FGF2 were withdrawn. Human recombinant NT3 (5ng/μl, R&D Systems) was added to enhance cell survival.

Quantitative real-time PCR (qPCR)

RNA was extracted using the GenElute Mammalian Total RNA Miniprep Kit and the On-Column DNAse I Digestion Set (Sigma-Aldrich). cDNA synthesis was performed with the Maxima First Strand cDNA Synthesis Kit (Thermo Fisher Scientific). Applied Biosystems SYBR Green PCR Master Mix was used for qPCR. Samples were run on the Applied Biosystems 7500 fast PCR machine. All samples were analyzed in technical duplicates and normalized to the house-keeping gene Porphobilinogen Deaminase 1 (*PBGD*). Results were analyzed with the ΔΔCt method. See supplemental experimental procedures for a full list of primer sequences.

Flow cytometry

For analysis of EGFP expression cells were harvested with TrypLE Select (Gibco) for 5-10 minutes at 37°C to obtain a single cell suspension. Following a wash with PBS, cells were resuspended in ice-cold PBS supplemented with DAPI (10μg/ml), and incubated for 5 minutes on ice. Cells were analyzed using a Cyan ADP flow-cytometer to determine the levels of EGFP expression of viable cells (DAPI negative). EGFP levels in non-induced (not dox-treated) iEGFP cells were indistinguishable from wild-type cells and served as negative control. FITC (EGFP) acquisition settings were set to enable plotting of the highest levels of EGFP expression. Thus, the negative control (wild-type and/or uninduced iEGFP cells) is in some instances located directly adjacent to the left y-axis. For staining and analysis of myosin heavy chain expression, cells were harvested with TrypLE Select (as for EGFP expression analysis), washed once with PBS, and fixed and permeabilized with Cytofix/Cytoperm solution (BD Biosciences). Subsequently, cells were washed and blocked in Perm/Wash buffer (BD Biosciences) supplemented with 3% bovine serum albumin (BSA) at +4°C overnight. Staining with a PE-conjugated anti-MYH antibody (supplemental experimental procedures) was carried out in Perm/Wash buffer for 1h at +4°C in the dark. After three washes with Perm/Wash buffer cells were analyzed with a Cyan ADP flow-cytometer to determine the levels of MHC expression. Induced myocytes stained with an IgG2b isotype-control antibody served as negative control for gating purposes. Stained non-induced iMYOD1 OPTi-OX hPSCs served as experimental negative control. Data analysis was performed with FlowJo (v10) and Graphpad Prism (v6).

Western blot

Whole-cell protein was extracted with CelLytic M (Sigma-Aldrich) supplemented with complete Protease Inhibitor (Roche), and subsequently quantified by using Protein Quantification Kit-Rapid (Sigma-Aldrich). Protein electrophoresis was performed with NuPAGE LDS Sample Buffer and 4-12% NuPAGE Bis-Tris Precast Gels (Invitrogen). Following protein transfer on PVDF, membranes were blocked with PBS supplemented with 0.05% Tween-20 (PBST) 4% milk for 1h at room temperature, and incubated with primary antibodies overnight in PBST 4% milk. Membranes were washed with PBST, incubated with HRP-conjugated secondary antibodies (Sigma-Aldrich) in PBST 4% milk, incubated with Pierce ECL2 Western Blotting Substrate (Thermo Fisher Scientific), and exposed to X-Ray Super RX Films (Fujifilm).

Immunocytochemistry

Cells were fixed in 4% paraformaldehyde (diluted in PBS) for 20 minutes at room temperature and subsequently washed three times with PBS. The cells were then blocked with 10% donkey serum (Sigma-Aldrich) and permeabilized with 0.3% Triton X-100 (diluted in PBS) for 20 minutes at room temperature. Subsequently, cells were incubated with appropriately diluted primary antibodies (supplemental experimental procedures) in 2% donkey serum and 0.1% Triton X-100 (diluted in PBS) at 4°C overnight. Triton-X was omitted throughout all steps when staining the surface antigen PDGFRA, A2B5, and O4. After three washes with PBS, the cells were incubated for 1 hour at room temperature with corresponding donkey fluorophore-conjugated secondary antibodies (Alexa Fluor 488, 555, 568, and/or 647) in PBS supplemented with 1% donkey serum. Nuclei were visualized with

4',6-diamidino-2-phenylindole (DAPI, Thermo Fisher Scientific). EGFP expression and immunostainings were imaged using a Zeiss LSM 700 confocal microscope (Leica). The percentage of βIII-tubulin positive cells was calculated by determining βIII-tubulin expression in at least 50 randomly selected DAPI-positive cells in 3 visual fields of 3 biological replicates using an inverted Olympus IX71 fluorescence microscope.

Statistical analysis

Statistical analysis was performed with GraphPad Prism (v6). The number of replicates, the statistical test used, and the test results are described in the figure legends. Unless stated otherwise data is presented as mean ± SEM.

List of primers for genotyping PCR

Locus	PCR type	Primer binding site	Primer sequence
hROSA26	Locus PCR	Genome (5')	GAGAAGAGGCTGTGCTTCGG
		Genome (3')	ACAGTACAAGCCAGTAATGGAG
	5'-INT PCR	Genome (5')	GAGAAGAGGCTGTGCTTCGG
		Splice Acceptor	AAGACCGCGAAGAGTTTGTCC
	3'-INT PCR	rtTA	GAAACTCGCTCAAAAGCTGGG
		Genome (3')	ACAGTACAAGCCAGTAATGGAG
	3'-BB PCR	rtTA	GAAACTCGCTCAAAAGCTGGG
		Vector Backbone (3')	TGACCATGATTACGCCAAGC
AAVS1	Locus PCR	Genome (5')	CTGTTTCCCCTTCCCAGGCAGGTCC
		Genome (3')	TGCAGGGGAACGGGGCTCAGTCTGA
	5'-INT PCR	Genome (5')	CTGTTTCCCCTTCCCAGGCAGGTCC
		Puromycin	TCGTCGCGGGTGGCGAGGCGCACCG
	3'-INT PCR	Transgene	transgene specific sequence
		Genome (3')	TGCAGGGGAACGGGGCTCAGTCTGA
	3'-BB PCR	Transgene	transgene specific sequence
		Vector Backbone (3')	ATGCTTCCGGCTCGTATGTT

List of primers for quantitative PCR

Gene	Orientation	Primer sequence
CNP	Fw	TCCTCATCATGAACAGAGGCTT
	Rev	AAACTGCAGCTCAGGCTTGT
CSPG4 (NG2)	Fw	GTCGAGGACACCTTCCGTTT
	Rev	GTGGTCAGCAGAGAGGACAC
DES	Fw	CCAACAAGAACAACGACGCC
	Rev	ATCAGGGAATCGTTAGTGCCC
DMD	Fw	TGGTGGGAAGAAGTAGAGGACT
	Rev	TGCTGCTTCCCAAACTTAGA
EGFP	Fw	CCCGACAACCACTACCTGAG
	Rev	GTCCATGCCGAGAGTGATCC
FOXG1	Fw	TGCCAAGTTTTACGACGGGA
	Rev	GGGTTGGAAGAAGACCCCTG
GRIA4	Fw	GGCCAGGGAATTGACATGGA
	Rev	AACCAACCTTTCTAGGTCCTGTG
HMBS (PBGD)	Fw	ATTACCCCGGGAGACTGAAC
	Rev	GGCTGTTGCTTGGACTTCTC
MAG	Fw	CAGAAGACGTCCCCAACTCA
	Rev	CCTCGGGAGGCTGAAATCATAA
MAP2	Fw	AGACTGCAGCTCTGCCTTTAG

	Rev	AGGCTGTAAGTAAATCTTCCTCC
MBP	Fw	TGGTGATGGAGATGTCAAGCAGGT
	Rev	GCTGTGGTTTGGAAACGAGGTTGT
MOG	Fw	AGAGATAGAGAATCTCCACCGGA
	Rev	TGATCAAGGCAACCAAGGGTC
MYH1	Fw	CACACTAGTTTCACAGCTCTCG
	Rev	CAGGGCACTCTTGGCCTTTA
MYH2	Fw	GGAAGCTCTGGTGTCTCAGTT
	Rev	CAGGGCGTTCTTGGCTTTTAT
MYH3	Fw	GCTGCATACCCAGAACACCA
	Rev	CCCTGCTGGCATCTTCTACC
MYH4	Fw	TCGCATTTGTCAGCCAAGGG
	Rev	TGAAACCCAGGATGTCCACAG
MYH7	Fw	GAGACTGTCGTGGGCTTGTA
	Rev	GCCCTTCTCAATAGGCGCATC
MYH8	Fw	TGAAGCAGATAGCAGCGCGA
	Rev	CGTACGAAGTGAGGGTGTGT
MYOD1 (endo)	Fw	GCCGCTTTCCTTAACCACAA
	Rev	CTGAATGCCCACCCACTGTC
MYOD1	Fw	CGACGGCATGATGGACTACA
	Rev	TAGTAGGCGCCTTCGTAGCA
NANOG	Fw	AGCAGATGCAAGAACTCTCCAA
	Rev	TGAGGCCTTCTGCGTCACAC
NEUROG2 (NGN2)	Fw	TGTTCGTCAAATCCGAGACCT
	Rev	CGATCCGAGCAGCACTAACA
NKX2.2	Fw	GCTTCCTGCGTCCATTTCCG
	Rev	GAAAGAAACTGGGGATGGGGAG
OLIG1	Fw	TGTCGCAGAGAGTTTTCGCT
	Rev	ATGCAAGGCGGTTGGTTTTC
OLIG2	Fw	ATCGCATCCAGATTTTCGG
	Rev	CCCCAGGGGAAGATAGTCGT
PAX6	Fw	CGAGATTTCAGAGCCCCATA
	Rev	AAGACACCACCGAGCTGATT
PDGFRA	Fw	AGGGATAGCTTCCTGAGCCA
	Rev	AGCTCCGTGTGCTTTCATCA
PLP	Fw	AACAGCTGAGTTCCAAATGACC
	Rev	ACGGCAAAGTTGTAAGTGGC
POU3F2 (BRN2)	Fw	ACCCGCTTTATCGAAGGCAA
	Rev	CCTCCATAACCTCCCCCAGA
POU5F1 (OCT4)	Fw	GTGGAGGAAGCTGACAACAA
	Rev	ATTCTCCAGGTTGCCTCTCA
RYR1	Fw	CAATCGCCAGAACGGAGAGA
	Rev	GTCGTGTTCCCTGTCTGTGT
SLC17A6 (VGLUT2)	Fw	GTAGACTGGCAACCACCTCC
	Rev	CCATTCCAAAGCTTCCGTAGAC
SOX10	Fw	ACACCTTGGGACACGGTTTT
	Rev	GTCCAACGCCCACCTCC
SYP	Fw	ACCTCGGGACTCAACACCTCGG
	Rev	GAACCACAGGTTGCCGACCCAG
SYN1	Fw	CCCTGGGTGTTTGCCCAGAT
	Rev	ACCACGGGGTACGTTGTACT
TUBB3	Fw	CAACCAGATCGGGGCCAAGTT
	Rev	CCGAGTCGCCCACGTAGTT

List of antibodies

Antigen	Species	Isotype	Clonality	Company	Cat. No.	Dilution
A2B5	mouse	IgM	monoclonal	Millipore	MAB312	1:300
ACTN2 (α-actinin)	mouse	IgG1	monoclonal	Sigma	A7811	1:200
BrdU	mouse	IgG1	monoclonal	BD Bio	347580	1:100
CNP	mouse	IgG1	monoclonal	Abcam	ab6319	1:500
DES (desmin)	rabbit	IgG	monoclonal	Abcam	ab32362	1:500
EOMES	rabbit	IgG	polyclonal	Abcam	ab23345	1:200
MAG	mouse	IgG1	monoclonal	Abcam	ab89780	1:400
MAP2	mouse	IgG1	monoclonal	Sigma	M4403	1:200
MBP	rat	IgG2a	monoclonal	Millipore	MAB386	1:200
MYOD1	rabbit	IgG	monoclonal	Abcam	ab133627	1:250
MYOG (myogenin)	mouse	IgG1	monoclonal	DSHB	F5D	1:100
MYOG (myogenin)	rabbit	IgG	monoclonal	Abcam	ab124800	1:500
MYH (myosin heavy chains)	mouse	IgG2b	monoclonal	DSHB	MF20	1:100
MYH-PE	mouse	IgG2b	monoclonal	BD Biosc.	564408	1:20 (Flow)
NANOG	goat	IgG	polyclonal	R&D	AF1997	1:200
NCAM	mouse	IgG1	monoclonal	DSHB	5.1H11	1:100
NEFH (neurofilament)	rabbit	IgG	polyclonal	Abcam	ab8135	1:1000
NEUROG2 (NGN2)	rabbit	IgG	Polyclonal	Santa Cruz	sc-50402	1:100
NKX2.5	rabbit	IgG	polyclonal	Santa Cruz	sc14033	1:200
O4	mouse	IgM	monoclonal	R&D	MAB1326	1:1000
OCT4	mouse	IgG2b	monoclonal	Santa Cruz	sc5279	1:200
PAX3	mouse	IgG2a	monoclonal	DSHB	Pax3	1:100
PAX6	mouse	IgG1	monoclonal	DSHB	PAX6	1:100
PAX7	mouse	IgG1	monoclonal	DSHB	PAX7	1:100
PLP	rabbit	IgG	monoclonal	Abcam	Ab183493	1:2000
SLC17A7 (VGLUT1)	goat	IgG	polyclonal	Abcam	ab104899	1:500
TNNT2 (troponin T)	mouse	IgG2a	monoclonal	DSHB	CT3	1:100
TTN (titin)	mouse	IgM	monoclonal	DSHB	9D10	1:100

TetR (tet repressor)	mouse	IgG1	monoclonal	Clontech	631131	1:1000 (WB)
TUBA4A (α4-tubulin)	mouse	IgG1	monoclonal	Sigma	T6199	1:10000 (WB)
TUBB3 (βIII-tubulin)	mouse	IgG1	monoclonal	Millipore	MAB1637	1:1000

Supplemental References

Cheung, C. et al., 2012. Generation of human vascular smooth muscle subtypes provides insight into embryological origin–dependent disease susceptibility. *Nature Biotechnology*, 30(2), pp.165–173.

Irion, S. et al., 2007. Identification and targeting of the ROSA26 locus in human embryonic stem cells. *Nature biotechnology*, 25(12), pp.1477–82.

Ladewig, J. et al., 2012. Small molecules enable highly efficient neuronal conversion of human fibroblasts. *Nature methods*, 9(6), pp.575–8.

Pang, Z.P. et al., 2011. Induction of human neuronal cells by defined transcription factors. *Nature*, 476(7359), pp.220–3.

Touboul, T. et al., 2010. Generation of functional hepatocytes from human embryonic stem cells under chemically defined conditions that recapitulate liver development. *Hepatology*, 51(5), pp.1754–65.

Vallier, L., 2011. Serum-free and feeder-free culture conditions for human embryonic stem cells. *Methods in molecular biology*, 690, pp.57–66.

Zhou, X. et al., 2006. Optimization of the Tet-On system for regulated gene expression through viral evolution. *Gene therapy*, 13(19), pp.1382–1390.
