# Supplementary figures and images for: Inducible and Deterministic Forward Programming of Human Pluripotent Stem Cells into Neurons, Skeletal Myocytes, and Oligodendrocytes
Source: Stem Cell Reports. 2017 Mar 23;8(4):803–12. doi: 10.1016/j.stemcr.2017.02.016 (PMC5390118; doi:10.1016/j.stemcr.2017.02.016)

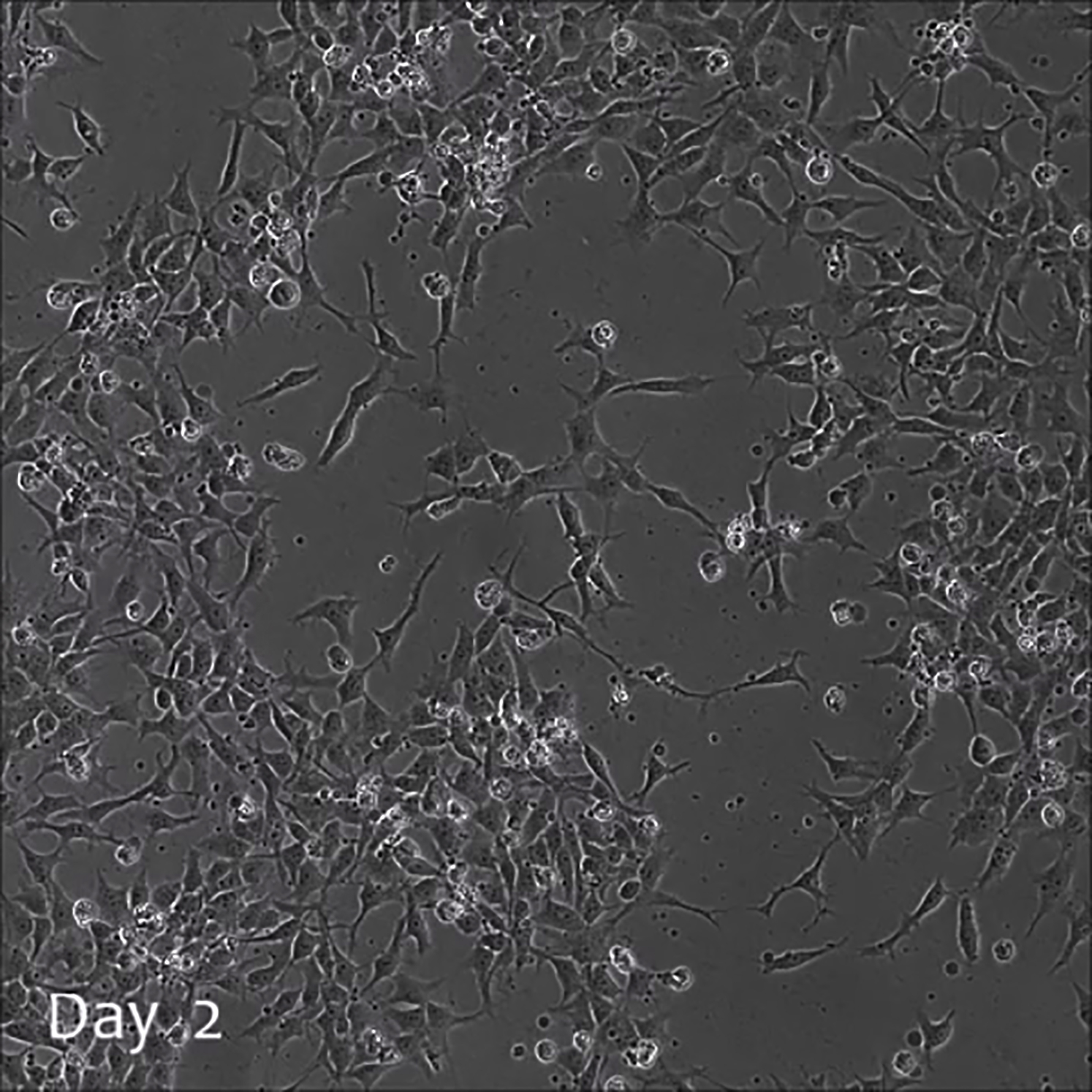

Supplement: Movie S1. Time-Lapse of Neuron Induction, Related to Figure 2 [file mmc2.jpg]

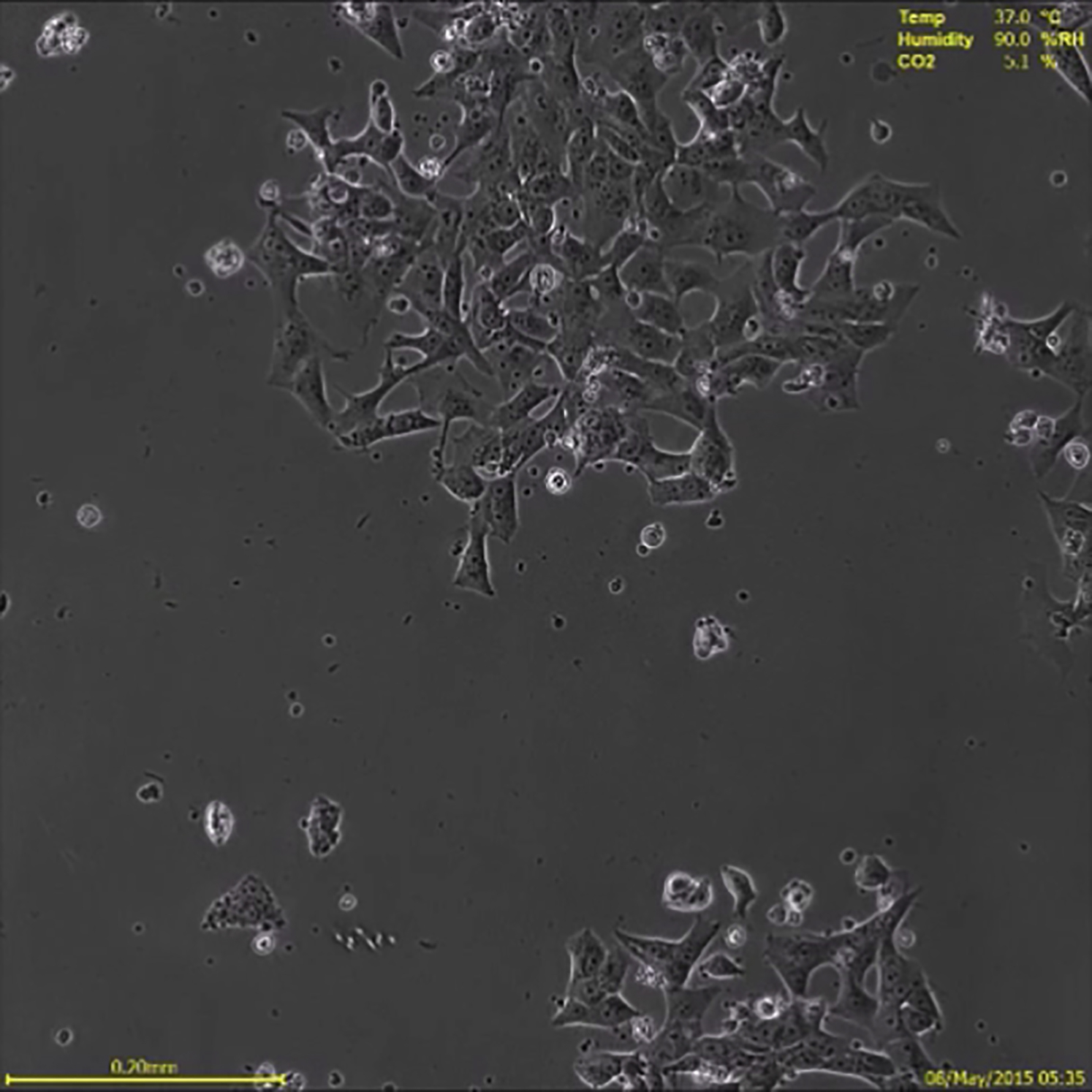

Supplement: Movie S2. Time-Lapse of Myocyte Induction, Related to Figure 3 [file mmc3.jpg]

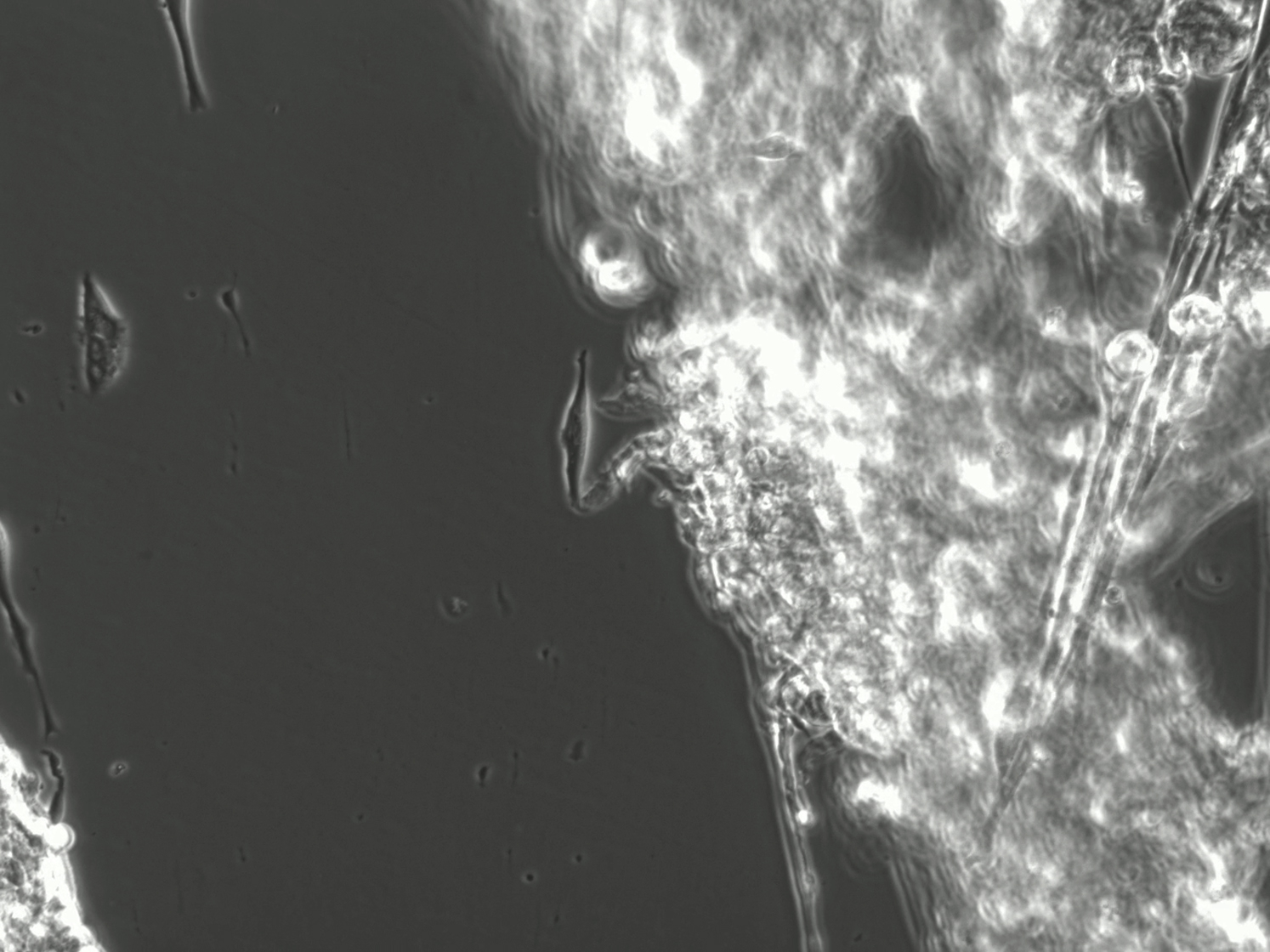

Supplement: Movie S3. Response of Induced Myocytes to ACh-Receptor Stimulation, Related to Figure 3 [file mmc4.jpg]
